# Multimodal perishable fruits and vegetables dataset

**DOI:** 10.1016/j.dib.2026.112545

**Published:** 2026-02-06

**Authors:** Devika Unnikrishnan, Krishna Deepak, Yogini Aishwaryaa P T S, Bagyammal T

**Affiliations:** Department of Computer Science and Engineering, Amrita School of Computing, Coimbatore, Amrita Vishwa Vidyapeetham, India

**Keywords:** Fruits classification, Computer vision, Machine learning, Thermal image, Methane gas sensor, Shelf-life prediction, Spoilage detection, Sustainable agriculture, Agriculture 5.0, SDG 2, SDG 12, SDG 13

## Abstract

There is a growing need in the agricultural industry for non-invasive methods to classify the freshness and quality of produce. To address this, we developed a multimodal dataset comprising six commonly exported fruits and vegetables from India: guava, carrot, tomato, Indian gooseberry, banana, and mango. The specimens were allowed to undergo decomposition in an indoor environment with natural lighting, ambient temperature fluctuations and controlled air-flow. During this process, IR-Fusion images, sRGB images, and methane concentration readings were collected over a varying period and compiled. The dataset supports research in classification, food spoilage detection, shelf-life prediction, multimodal data fusion, non-invasive fruit quality assessment, and deep learning-based freshness assessment, particularly for export-oriented supply chains. The dataset is motivated by the need to reduce post-harvest losses and improve food quality monitoring, where spoilage indicators are often not detectable through visual inspection alone; the integration of imaging and gas-based sensing enables more reliable and automated freshness assessment. The dataset, with a total size of 18.99 GB, contains over 14,000 sRGB images, 14,500 IR-fusion images, and 18 methane sensor files, organized into Normal and Classified (Spoiled/Not_spoiled) categories. This multimodal design enables the study of thermal, visual, and chemical spoilage indicators simultaneously. This work aligns with the principles of smart agriculture, which promote the use of modern, data-driven technologies to optimize resource use and enable real-time monitoring for sustainable and efficient agricultural practices. Within the Agriculture 5.0 (AG 5.0) paradigm, the integration of Artificial Intelligence (AI), the Internet of Things (IoT), and complementary sensing modalities plays a central role in advancing innovation across farming and post-harvest management. In this context, the proposed multimodal dataset supports AG 5.0 objectives by enabling intelligent, automated, and non-invasive produce quality assessment, thereby improving decision-making and reducing waste throughout agricultural and export-oriented supply chains.

Specifications Table

**Table:** 

Subject	Computer Sciences
Specific subject area	Computer vision, Image processing, Image classification, Shelf-life prediction, Multimodal data quality inspection, Machine learning, IoT-based smart pattern recognition.
Type of data	The dataset contains sRGB images (JPG), IR-Fusion thermal images (JPG), and MQ4 methane measurements, including ADC and resistance data, stored in .txt files. Around 29,000 images (sRGB + IR-Fusion) and 18 methane sensor text files, amounting to multiple gigabytes of multimodal data.
Data collection	The data was collected using a Fluke TiS40 Thermal Imaging Camera, Samsung S23 Ultra, Samsung S23, Samsung A34, Arduino Uno and MQ4 gas sensor. Data was collected three times per day. For each collection instance, 17 thermal IR-Fusion images and 17 sRGB images were captured, along with two gas sensor readings from each side of the sample. The samples were allowed to be decomposed in an indoor environment. sRGB images, thermal IR-Fusion images and methane level readings of local export fruits that were allowed to decompose under room temperature in the Southern Indian Monsoon climate.
Data source location	The data was collected from Amrita Vishwa Vidyapeetham, Ettimadai, Coimbatore, Tamil Nadu, India, from 2025 to 06–25 to 2025–10–03.
Data accessibility	Repository name: TriModal Ripeness 6 Data identification number: 10.6084/m9.figshare.30783827 Direct URL to data: https://doi.org/10.6084/m9.figshare.30783827
Related research article	None

## 1 Value of the Data

• Comprehensive lifecycle coverage: The dataset documents the full lifecycle of fruits, from the unripe stage to the visible spoilage stage, using multimodal data. Measurements are captured three times daily—morning, afternoon, and evening—recording natural variations in temperature, color, and methane emission patterns.
• Multimodal Dataset: The dataset uniquely combines IR-Fusion images, sRGB images, and methane concentration readings, allowing correlation of thermal, visual, and chemical changes. This integration supports research into non-destructive fruit quality monitoring and spoilage analysis [[1], [2], [3]].
• Application in machine learning: The dataset can be used to train and validate machine learning models for ripeness detection, spoilage prediction [1,2], and shelf-life estimation, and automated fruit sorting and grading systems [4].
• Real-world variability and representativeness: Spoilage duration and observation counts vary across fruit types, reflecting natural differences in decay rates. This variability closely mirrors real-world conditions, improving model robustness, and generalization [5]. Within-class variability is critically important for deep learning because it prevents models from learning overly simplistic or spurious patterns and instead forces them to capture robust, generalizable features. In the context of early-stage spoilage, subtle and overlapping thermal gradients and visual cues introduce challenging intra-class variations. By capturing early-stage spoilage where fresh and spoiled samples exhibit similar thermal and visual characteristics, the dataset incorporates meaningful intra-class diversity.
• Reusability and extension potential: The dataset can be reused for time-series analysis, multimodal data fusion, and sensor–image correlation studies and can be extended with additional sensing modalities such as humidity or gas composition. The data is publicly available and accessible [6].

## 2 Background

The compilation of this dataset was motivated by the increasing need for non-invasive, multimodal approaches to assess the freshness and quality of agricultural produce more accurately. Nearly one-third of global food production—approximately 1.7 billion tons—is wasted each year, emphasizing the importance of reducing post-harvest and storage losses for long-term food security and sustainability [7]. Prior studies have discussed the effectiveness of thermal imaging in agriculture across various pre-harvest and post-harvest operations [3], while AI technologies such as predictive modelling, automated grading, and storage monitoring have shown potential in supporting continuous, data-driven quality assessment. Existing image-based datasets often lack integrated sensing modalities, and this dataset was therefore generated to provide a more comprehensive representation of the decomposition process in perishable items [4].

By supporting sustainability and resilience in agriculture, the proposed multimodal produce dataset contributes to key Sustainable Development Goals (SDG). It aligns with SDG 2 (Zero Hunger) by enabling improved monitoring of produce quality and early detection of spoilage, which supports food security and reduces losses in the supply chain. It also supports SDG 12 (Responsible Consumption and Production) by facilitating automated, non-invasive quality assessment that helps minimize post-harvest waste and promotes more efficient use of resources. Additionally, by providing data that can inform adaptive and efficient post-harvest management practices, the dataset indirectly contributes to SDG 13 (Climate Action) through the reduction of unnecessary waste and associated environmental impacts [8].

The data were collected in an indoor environment with natural lighting, ambient temperature fluctuations and controlled air-flow to support studies involving freshness estimation, decay detection, and automated classification using machine learning and deep learning techniques. The dataset also reflects the need for methods beyond traditional visual inspection, as many spoilage indicators are not externally visible. Thermal imaging captures temperature variations associated with microbial activity, bruising, and fermentation, while methane gas sensing provides chemical information related to anaerobic decomposition, enabling richer multimodal observations of spoilage on retail store shelves.

## 3 Data Description

**Fig. 1:**
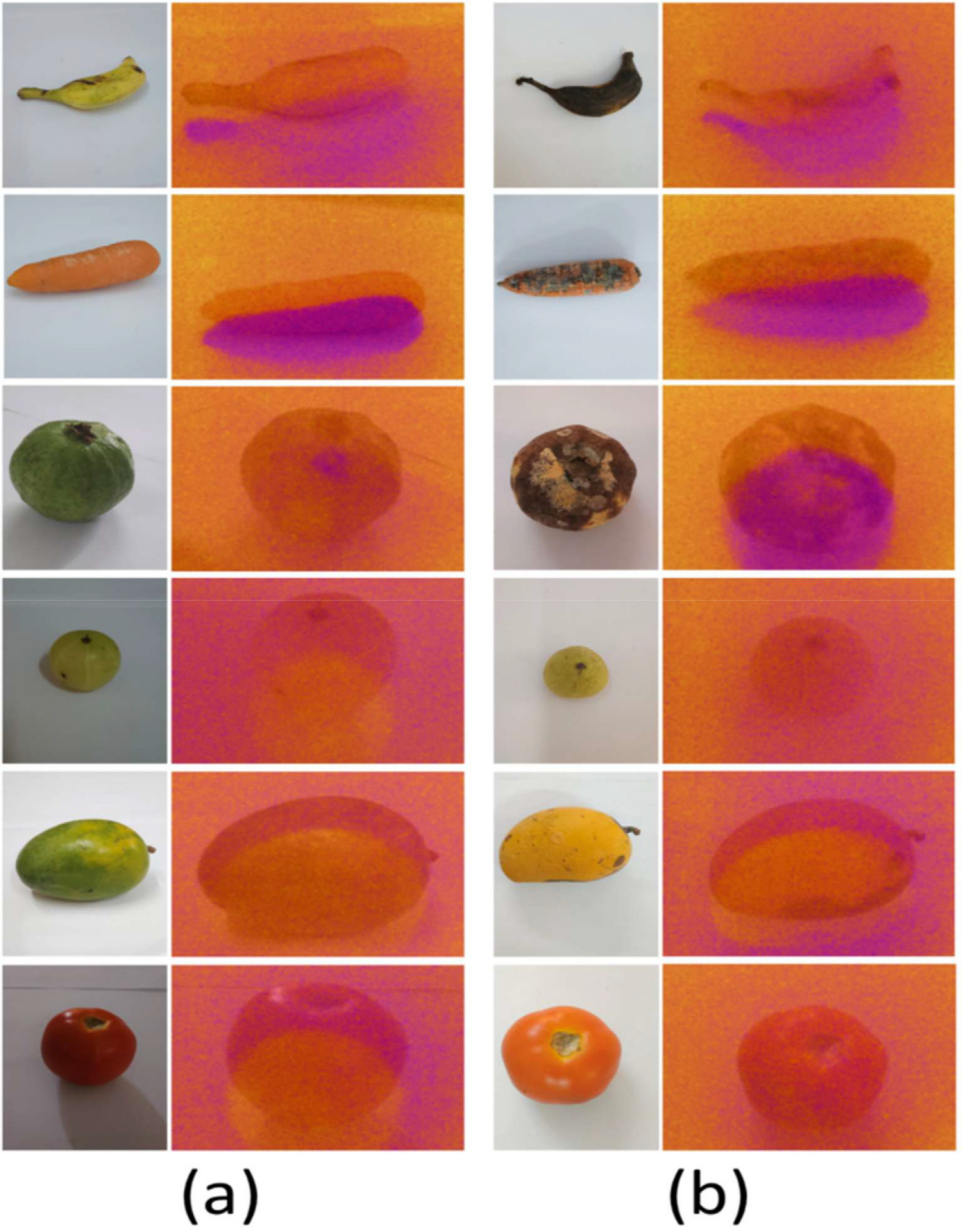
Sample sRGB and IR-Fusion images from various two quality categories: (a) Not_spoiled, (b) Spoiled.

**Fig. 2:**
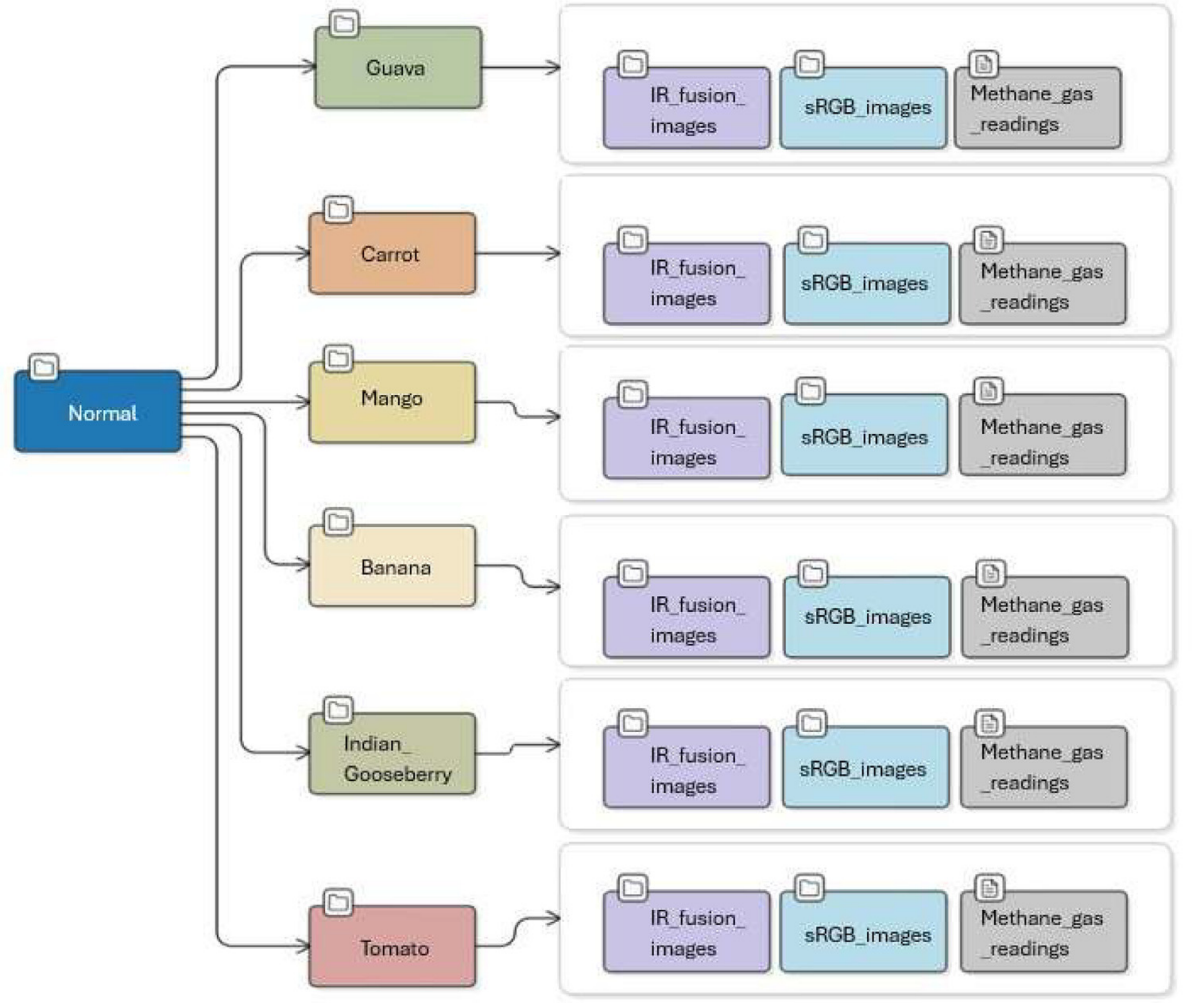
Structure of the data repository: Root folder is named as “TR-6” with subfolder Normal.

**Fig. 3:**
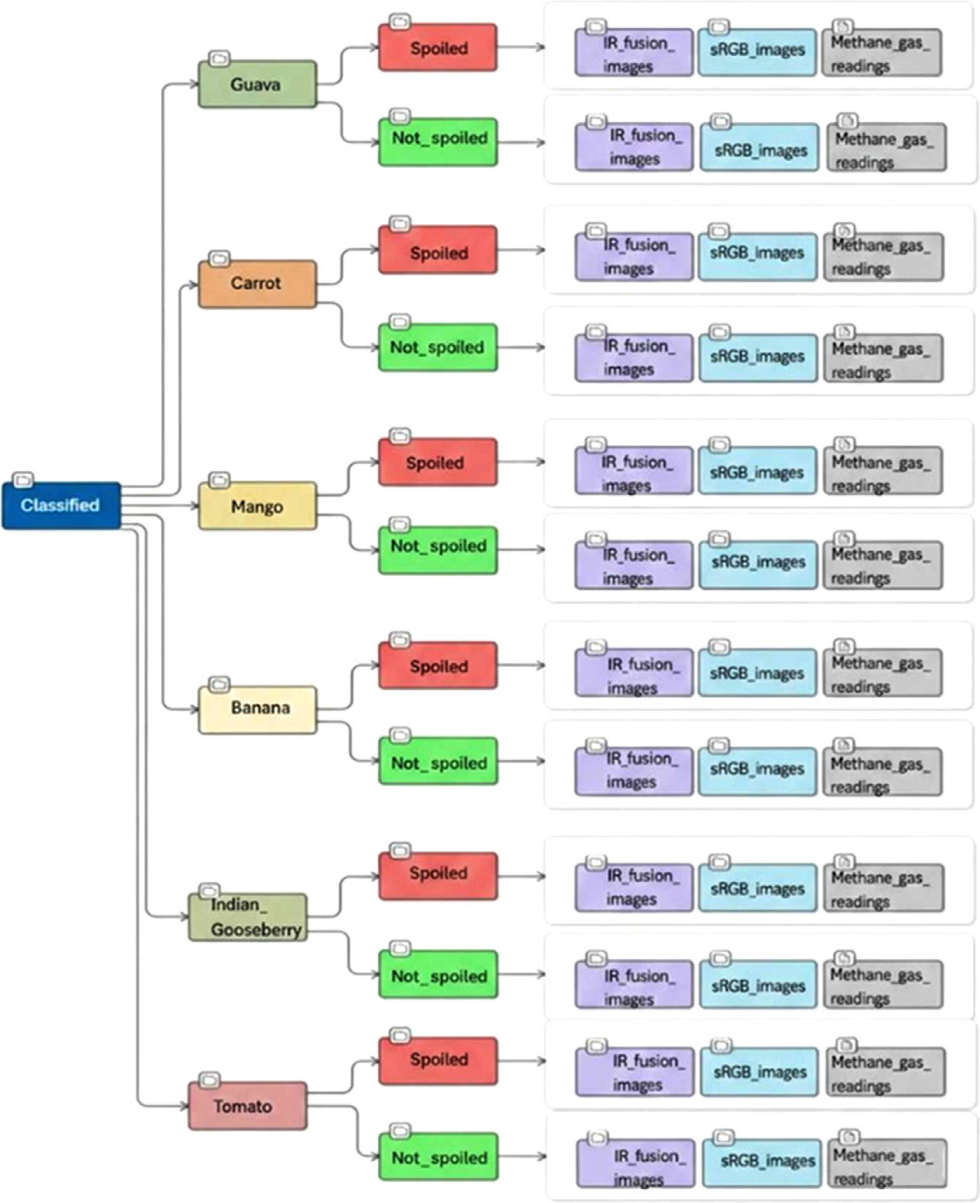
Structure of the data repository. The root folder is named as “TR-6” with subfolder Classified.

The dataset, Fig. 1, comprises three data modalities—Thermal IR-Fusion images, sRGB images, and methane-concentration readings—and is organized under a root directory named TR-6. The repository is divided into two main subfolders, Normal, Fig. 2, and Classified, Fig. 3, each containing the same six fruit categories: Guava, Carrot, Tomato, Indian_Gooseberry, Banana, and Mango. Each fruit-specific folder includes the complete set of files associated with that sample, ensuring consistency across modalities.

**Fig. 4:**
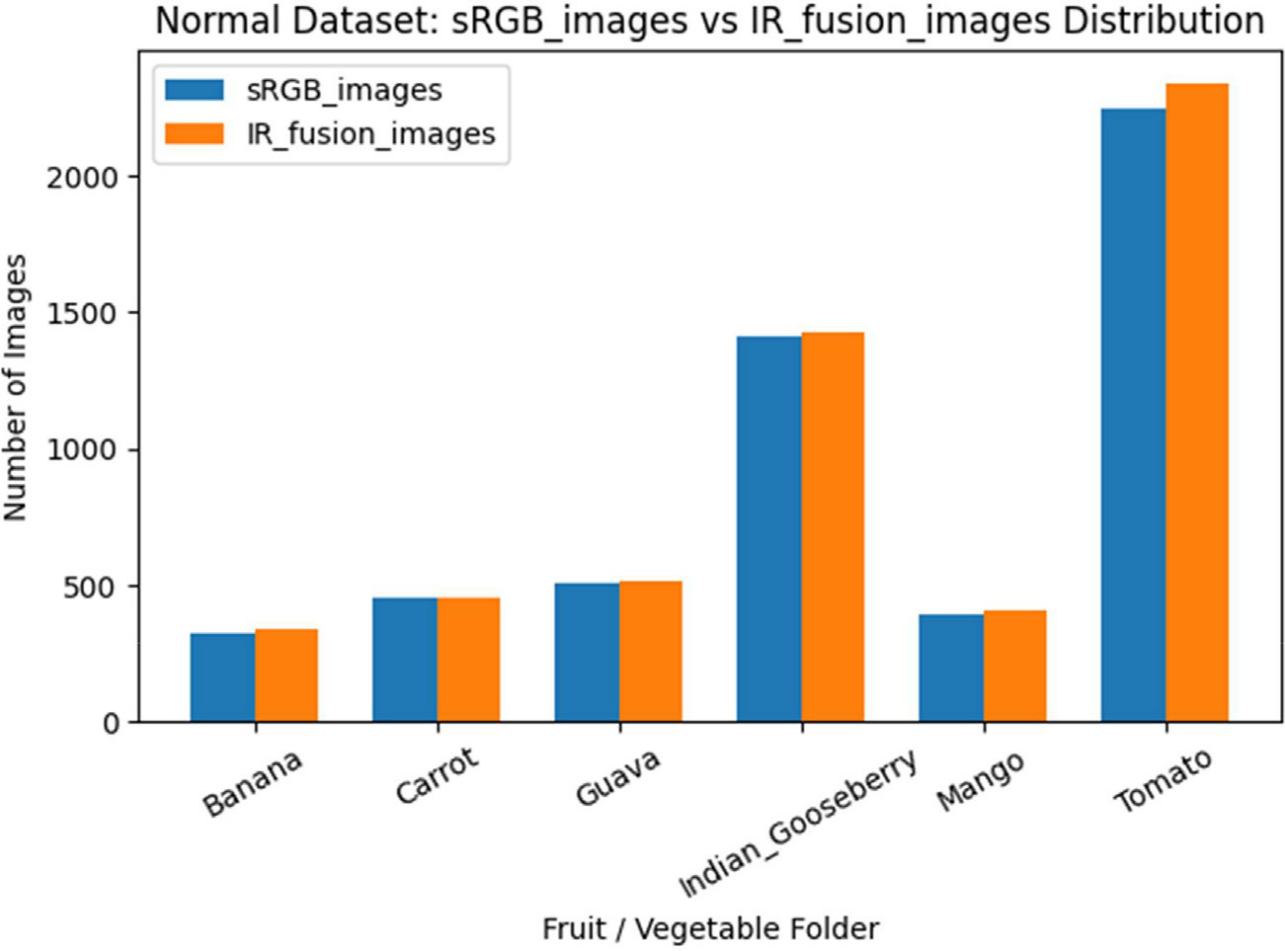
Distribution of sRGB and IR-Fusion images across fruit categories in the Normal dataset.

Within the Normal subfolder, the dataset contains a total of 5,338 sRGB images and 5,492 IR-Fusion images distributed across the six fruit categories, Fig. 4. Specifically, Banana contains 323 sRGB and 340 IR-Fusion images, Carrot contains 458 sRGB and 458 IR-Fusion images, Guava contains 510 sRGB and 518 IR-Fusion images, Indian Gooseberry contains 1,411 sRGB and 1,428 IR-Fusion images, Mango contains 392 sRGB and 407 IR-Fusion images, and Tomato contains 2,244 sRGB and 2,341 IR-Fusion images. In addition, each fruit category includes one methane-concentration text file, resulting in six methane sensor files in total for the Normal dataset. The IR_fusion_images folder stores thermal images in JPG format; for each fruit, 17 thermal images were captured per day, consisting of 16 side-view images acquired at equal angular intervals around the sample and one top-view image. Filenames include both the acquisition timestamp and the corresponding minimum and maximum temperature values. The sRGB_images folder contains sRGB images captured using smartphone cameras, stored in JPG format, with consistent positioning, framing, and acquisition conditions across all samples.

**Fig. 5:**
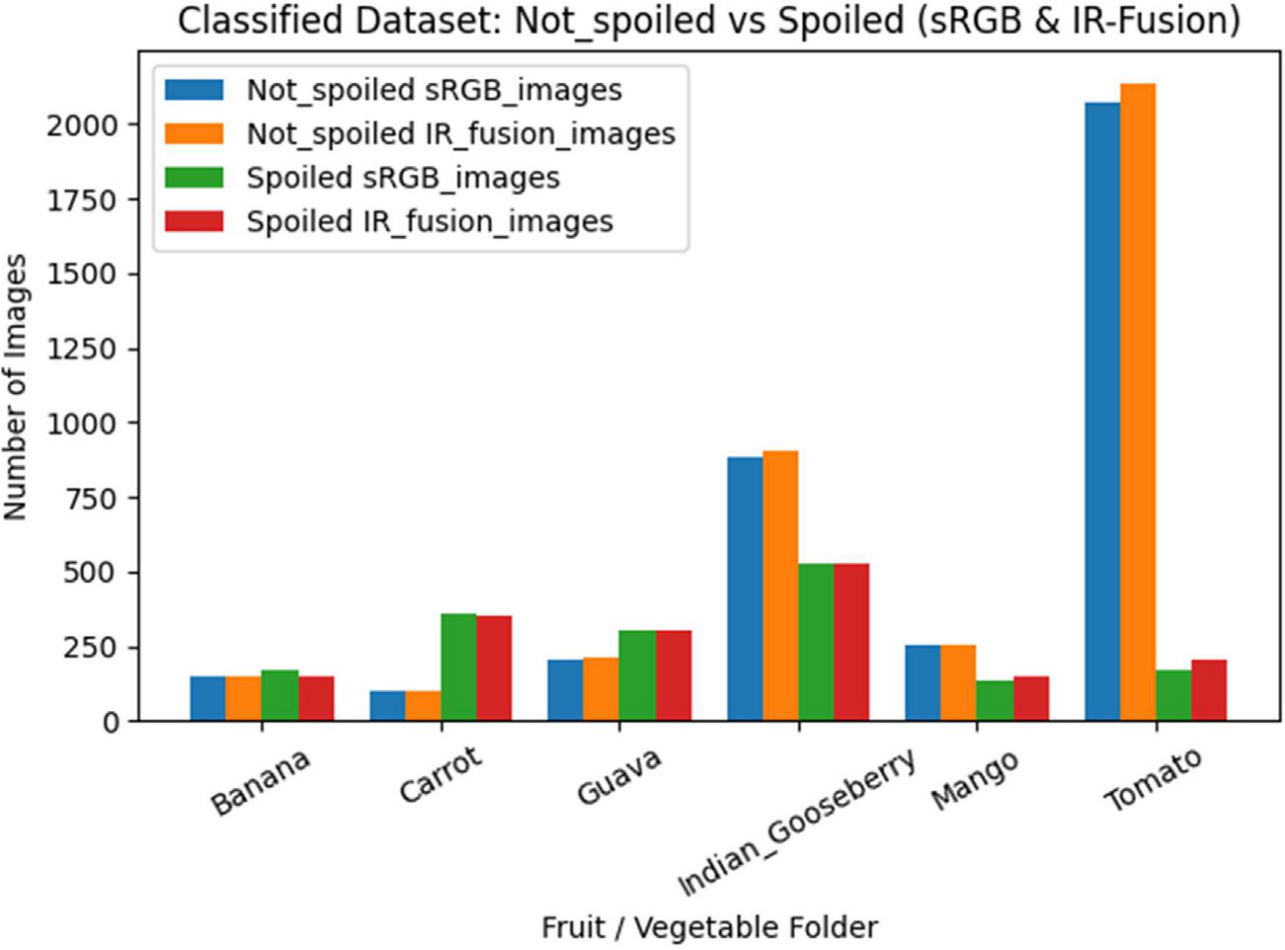
Distribution of sRGB and IR-Fusion images across fruit categories in the Classified dataset.

The Classified subfolder further separates the dataset into Not_spoiled and Spoiled categories and contains a total of 4,718 sRGB images and 4,808 IR-Fusion images, Fig. 5. In the Spoiled category, Banana includes 170 sRGB and 153 IR-Fusion images, Carrot includes 356 sRGB and 355 IR-Fusion images, Guava includes 306 sRGB and 306 IR-Fusion images, Indian Gooseberry includes 527 sRGB and 525 IR-Fusion images, Mango includes 136 sRGB and 152 IR-Fusion images, and Tomato includes 170 sRGB and 204 IR-Fusion images. In the Not_spoiled category, Banana includes 153 sRGB and 153 IR-Fusion images, Carrot includes 102 sRGB and 103 IR-Fusion images, Guava includes 204 sRGB and 212 IR-Fusion images, Indian Gooseberry includes 884 sRGB and 903 IR-Fusion images, Mango includes 256 sRGB and 255 IR-Fusion images, and Tomato includes 2,074 sRGB and 2,137 IR-Fusion images. Each classified fruit category includes one corresponding methane-concentration text file, yielding a total of twelve methane sensor files across the Classified dataset.

Within-class variability is observed in both thermal and visual modalities, particularly for early-stage spoilage where thermal gradients and visual cues are subtle and overlap with those of fresh samples.

## 4 Experimental Design, Materials and Methods

**Fig. 6:**
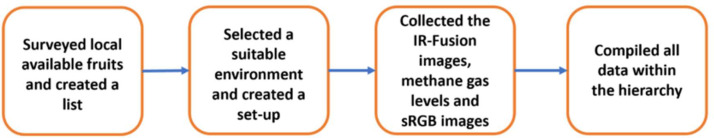
Data acquisition process.

**Fig. 7:**
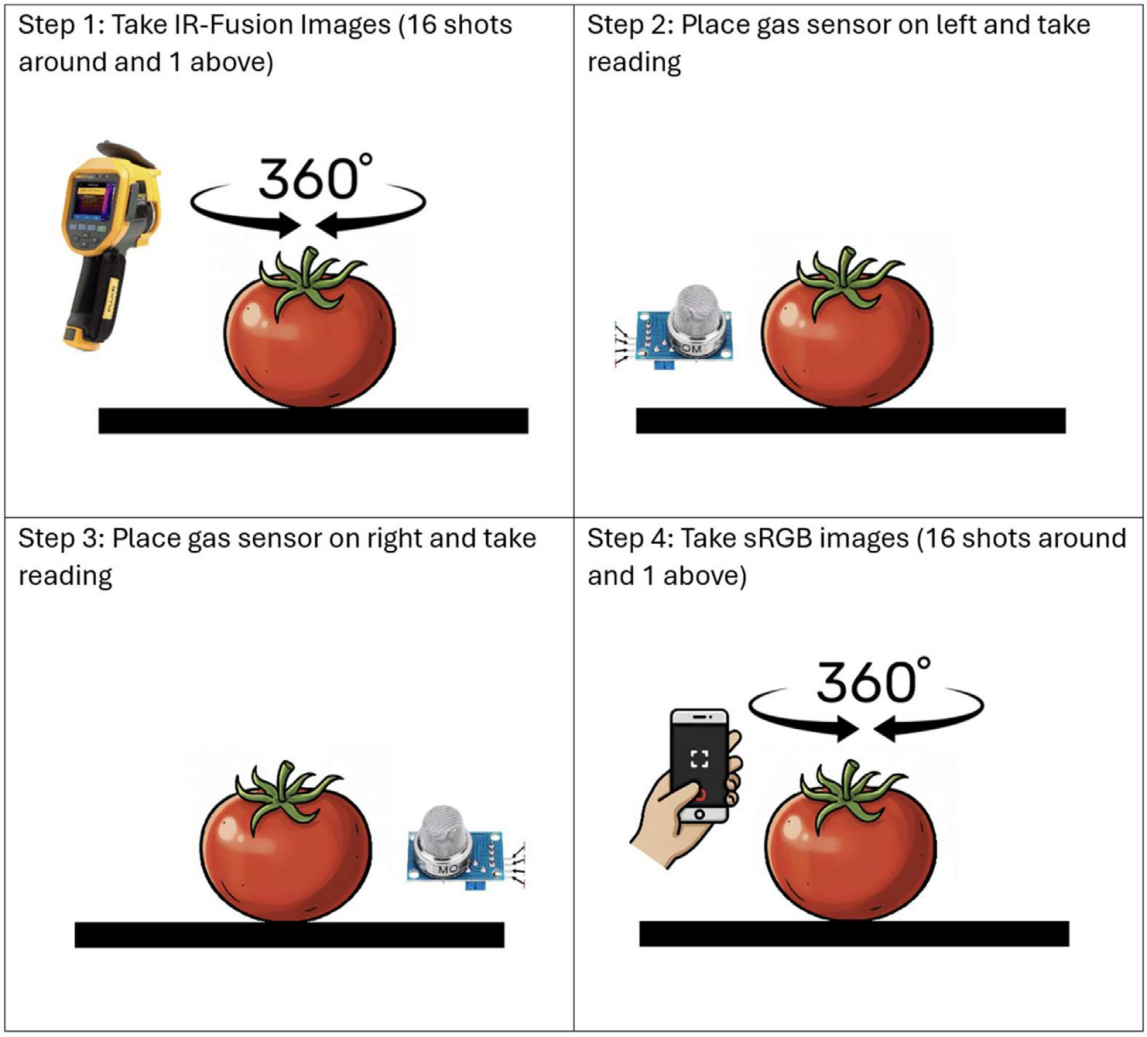
Data acquisition method.

Data acquisition was performed three times daily: in the morning, afternoon, and evening to capture variations in gas emissions and surface temperature throughout the day. The data acquisition flow is given in Fig. 6, and the method is given in Fig. 7. Each fruit was monitored daily until visible spoilage appeared, resulting in varying dataset durations across samples. The dataset contains IR fusion images, sRGB images, and gas readings for each day.

All experiments were conducted in an indoor room with minimal air disturbance. Doors and windows were typically kept closed during data acquisition and were opened occasionally between sessions for routine access. No active ventilation systems or fans were used during measurements to minimize airflow-induced variations in gas concentration. All collected data were manually verified for quality. Ambient room temperature and relative humidity were not continuously logged during data acquisition. Consequently, no explicit correction or normalization of MQ-4 methane sensor readings with respect to temperature or humidity variations was performed. The measurements therefore reflect sensor responses under natural indoor environmental conditions.

The experiments were conducted in a closed room under natural lighting conditions. Fruits were placed individually on 5 standard A4 sheets placed on the table's surface. Each sample was monitored from the point of purchase until it exhibited visible signs of spoilage, such as surface darkening and fungal growth.

For each data acquisition session, fruits were placed at a fixed location on the table. The thermal camera was operated by hand, and care was taken to maintain an approximately consistent distance from the fruit across all samples and sessions. For each fruit, 16 images were captured from side-view angles evenly distributed around the fruit and one image was captured from a top-down view using the thermal camera; the same viewing strategy was applied for sRGB image acquisition.

Methane gas measurements were obtained by positioning the MQ-4 sensor alternately on the left and right sides of the fruit at the same vertical level, and readings from both positions were recorded for each session. This protocol was repeated three times daily for each fruit sample until visible spoilage was observed.

### 4.1 Thermal image acquisition

A Fluke TiS40 Thermal Imaging Camera, as mentioned in Table 1, was used to capture IR-fusion images by hand. For each fruit, 17 images were taken per day: 16 side-angle images evenly spaced around the fruit and one top-down image at 75% preset in AutoBlend mode. Surface temperature distributions and temporal variations in temperature patterns were recorded for each fruit.

**Table 1:** IR-fusion camera details and particulars.

Model	Tis40
Temperature range	−20 °C to 350 °C (−4°F to 662°F)
IFOV (Spatial resolution) Distance to Spot	3.9 mRad, D:S 275:1
Image capture frequency	9 Hz or 30 Hz refresh rate
Thermal sensitivity (NETD)	≤90 mK
Total pixels	160×120
Infrared spectral band	7.5 µm to 14 µm (long wave)
File Formats	BMP, JPG, IS2, IS3, AVI

### 4.2 sRGB image acquisition

Standard RGB images were captured and stored in JPG format using a Samsung Galaxy S23 Ultra smartphone, a Samsung Galaxy S23 smartphone, and a Samsung Galaxy A34 smartphone, as mentioned in Table 2, maintaining approximately the same orientation for consistency. Surface color changes, darkening and bruising, visible fungal growth, and morphological changes due to spoilage were recorded for each fruit.

**Table 2:** sRGB camera details and particulars.

	Galaxy S23 Ultra	Galaxy A34		Galaxy S23
Mode	Pro Mode	Pro Mode	Macro Mode	Pro Mode
Camera type	Smartphone	Smartphone	Smartphone	Smartphone
Smartphone type	Android	Android	Android	Android
Company name	Samsung	Samsung	Samsung	Samsung
Camera model	Sony IMX754	IMX582 Exmor RS	GalaxyCore GC5035	Samsung S5K3K1
F-stop	F1.8	F1.8	F2.4	F2.4
Focal length	69 mm	25 mm	25 mm	69 mm
Metering mode	Centre-weighted metering	Spot metering	Auto	Centre-weighted
Mode of flash	No flash	No flash	No flash	No flash
Resolution	3000×4000	3000×4000	1932×2576	3000×4000
Colour space	sRGB	sRGB	sRGB	sRGB
Megapixel count	12MP	12MP	5MP	12MP

### 4.3 Methane gas measurement

Methane emissions were recorded using an MQ4 methane gas sensor, interfaced with an Arduino Uno. The clean-air baseline resistance *R*_0_ of the MQ-4 sensor was determined once during the initial system setup. The sensor was operated in indoor clean air and allowed to warm up and stabilize for a period of approximately 3–4 h. During this interval, raw ADC values and corresponding sensor resistance *R_s_* were continuously monitored [9]. The value of *R*_0_ was computed after stabilization by averaging *R*_s_ over the final portion of the warm-up period, and this baseline value was subsequently used for all methane concentration estimations in the dataset.

Gas readings were taken simultaneously with thermal image captures and included raw ADC values, sensor resistance, and methane concentrations in ppm. The gas readings were saved as .txt files and mapped to their respective fruit samples. The sensor resistance Rs was computed from the measured output voltage *V_out_* using the voltage divider relationship:

(1) $$R_{s}=R_{l}\frac{\left(V_{c}-V_{out}\right)}{V_{out}}$$

where *R*_s_ is the sensor resistance (kΩ°), *R_l_* is the load resistance (kΩ°), *V_c_* is the circuit supply voltage (5.0 V), *V_out_* is the analog output voltage corresponding to the ADC reading (V). The normalized sensor ratio was then determined as:

(2) $$ratio=\frac{R_{s}}{R_{0}}$$

Where *R*_0_ is the sensor resistance in clean air (calibration value). Methane concentration (in ppm) was calculated based on the logarithmic relation obtained from the MQ-4 calibration curve:

(3) $$\log_{10}\left(ppm\right)=a\log_{10}\frac{R_{s}}{R_{0}}+b$$

Where *a* and *b* are empirical constants derived from the MQ-4 datasheet. These features were selected to ensure that the dataset captures both thermal anomalies, visual indicators, and gas signatures of spoilage.

## Limitations

The experiment was conducted in a closed room with natural lighting and ambient temperature without active environmental control which may introduce variations in temperature and methane gas readings across sessions. The dataset size is limited due to the manual nature of data collection and variation in fruit shelf life.

## Ethics Statement

The data is available in public. No ethics approval needed for this study.

## CRediT authorship contribution statement

**Devika Unnikrishnan:** Software, Investigation, Validation, Data curation, Writing – original draft. **Krishna Deepak:** Software, Investigation, Validation, Data curation, Writing – original draft. **Yogini Aishwaryaa P T S:** Software, Investigation, Validation, Data curation, Writing – original draft. **Bagyammal T:** Conceptualization, Methodology, Software, Supervision, Writing – review & editing.

## Data Availability

FigshareTriModal Ripeness 6 (Original data).
